# First Detection of International High-Risk *bla*_KPC-2_-Harbouring *Escherichia coli* Pandemic Lineage ST648 in Pet Food Packages

**DOI:** 10.1155/2024/9995914

**Published:** 2024-03-05

**Authors:** Ikechukwu Benjamin Moses, Ághata Cardoso da Silva Ribeiro, Tiago Barcelos Valiatti, Fernanda Fernandes Santos, Rodrigo Cayô, Ana Cristina Gales

**Affiliations:** ^1^Laboratório Alerta, Division of Infectious Diseases, Department of Internal Medicine, Escola Paulista de Medicina, Universidade Federal de São Paulo—UNIFESP, Rua Pedro de Toledo, 781, São Paulo-SP, Brazil; ^2^Department of Applied Microbiology, Faculty of Sciences, Ebonyi State University, P.M.B. 053, Abakaliki, Nigeria; ^3^Laboratory of Immunology and Microbiology (LIB), Division of Molecular Biology, Microbiology and Immunology, Department of Biological Sciences (DCB), Institute of Environmental, Chemical and Pharmaceutical Sciences (ICAQF), Universidade Federal de São Paulo (UNIFESP), Diadema-SP, Brazil

## Abstract

The continued worldwide increase in pet ownership has significantly boosted the growth of the pet food industry accompanied by new food safety risks and challenges. This study was designed to determine the occurrence and molecularly characterize multidrug-resistant (MDR) *Enterobacterales* in pet food. Eighty-six (86) packages of dry and wet pet food purchased in different retail stores were screened for carbapenem-resistant *Enterobacterales* (CRE). Antimicrobial susceptibility testing was performed by agar dilution technique using EUCAST/BrCAST recommendations. Blue-Carba test was further used to screen for carbapenemase-producing isolates. Isolated CRE strains were identified at the species level using matrix-assisted laser desorption/ionisation time-of-flight mass spectrometry (MALDI-TOF MS). Detection of carbapenemase-encoding genes was carried out by PCR, Sanger sequencing, and whole genome sequencing (WGS). A total of 15 (17.4%) MDR-CRE (*Escherichia coli* (*n* = 2), *Enterobacter cloacae* (*n* = 10), *Leclercia adecarboxylata* (*n* = 2), and *Cronobacter* spp. (*n* = 1)) were isolated from 86 pet food samples. In addition to being resistant to beta-lactams, the Gram-negative bacterial isolates were also resistant to aminoglycosides, fluoroquinolones, and tigecycline. Interestingly, two carbapenem-resistant *E. coli* isolates harboured *bla*_KPC-2_ gene. WGS analysis of the two *bla*_KPC-2_-producing *E. coli* isolates revealed that they both belong to ST648 and serotype O153:H2 group. The genetic context of the *bla*_KPC-2_ showed that they were carried by an IncN plasmid on a Tn*4401b* transposon element. To the best of our knowledge, this is the first description of *bla*_KPC-2_-harbouring *E. coli* ST648 pathogens in pet food. The detection of *bla*_KPC-2_-harbouring *E. coli* ST648 pandemic high-risk lineage in pet food is worrisome and a serious “One Health” issue. Therefore, pet food should be considered as a potential vehicle for the transmission of MDR pathogens to companion animals, and a risk factor for the dissemination of these bacterial pathogens to pet animals and their human guardians.

## 1. Introduction

The increasing global spread of antimicrobial resistance (AMR) is a critical issue that is no longer restricted to hospital settings, but also represents an important and underestimated growing problem involving food safety [1]. In the last decade, the frequency of pet ownership, especially dogs and cats, has significantly increased worldwide. This worldwide increase in pet ownership in recent years, especially in the United States with the largest pet population (223 million), followed by China (190 million), and Brazil (139.3 million) (http://petbrasil.org.br/importer), has been linked to an exponential growth of the pet food industry. This has been accompanied by new food safety risks such as AMR and pet food contamination by bacterial pathogens [2–4].

The worldwide spread of carbapenem-resistant *Enterobacterales* (CRE), including *bla*_KPC-2_-harbouring bacterial pathogens, is a serious concern and a challenging public health problem worldwide [5, 6]. Infections caused by CRE are associated with high morbidity and mortality rates due to the limited availability of therapeutic alternatives and the lackadaisical development of new antimicrobials [6]. The World Health Organization (WHO) considers CRE as global priority pathogens in critical need of next-generation antimicrobials and new control strategies [7, 8]. The five major carbapenemases identified in Gram-negative bacterial pathogens are those belonging to the groups KPC, NDM, VIM, IMP, and OXA-48 [9]. KPC-2 are endemic in low-and middle-income countries, especially in Latin America (such as Colombia, Brazil, Chile, Bolivia, and Argentina) and in some developed countries (such as USA and China) [10, 11].

Reports on pet food contamination by bacterial pathogens, especially CRE strains are very scarce as little or no attention has been paid to this surveillance in many countries and regions of the world, especially in Latin America, including Brazil. However, despite this neglect, Seiffert et al. [12] reported the presence of *bla*_OXA-48_ (13.3%) gene and other *β*-lactamase-encoding genes such as *bla*_CTX-M-15_ (53.3%), *bla*_CMY-4_ (20%), and *bla*_VEB-4-like_ (6.7%) in 30 packages of pet food samples obtained from three different retail outlets in Europe. The contamination of pet food by important foodborne pathogens has also been reported in a study carried out in the United States where 16.3%, 7.6%, and 4.1% of raw pet foods ordered online were positive for *Listeria monocytogenes*, *Salmonella* spp., and *Escherichia coli*, respectively [13].

Unlike the European Union and other countries that have implemented strict safety guidelines for pet food, e.g., the European Union Regulation (EC) No 142/2011 [14] and the USA Compliance Policy Guide Sec 690.800 in Food for Animals [15], Brazil and some other countries in the world are yet to establish strong guidelines on pet food safety standards. The established health safety guidelines in Brazil majorly focus on food-producing animals (livestock) and their products, and human health. In Brazil, the “Ministry of Agriculture and Livestock” oversees the inspection and supervision of products for animal feed, approval of technical regulations on hygienic-sanitary conditions and good manufacturing practices (GMPs) for establishments that manufacture animal feed or products intended for animal feed, and registration of pet food production companies; however, there are no well-defined standards/guidelines on pet (dogs and cats) food safety. The unavailability of well-defined guidelines on pet food safety standards in Brazil and other countries could contribute to the spread of antimicrobial resistance with significant public health impact, especially if contaminated pet food products are consumed by companion animals which are in close proximity with their human guardians.

The continued increase in AMR in pets, humans, and food-producing animals with their products has emerged as a leading public health threat in the 21st century [4]. Investigating the contamination of pet food by bacterial pathogens would be a major step in understanding the genesis of the zoonotic transmission of bacterial pathogens from companion animals to humans.

Herein, this study aimed to assess pet food as potential sources of carbapenemase-producing bacterial pathogens in the city of Sao Paulo, Brazil. To the best our knowledge, this study is the first to report the detection of *bla*_KPC-2_-harbouring *E. coli* ST648 in pet food in Brazil.

## 2. Materials and Methods

### 2.1. Sample Collection

Eighty-six (86) pet food package samples (dry and wet) belonging to 12 different brands were purchased from different retail stores in São Paulo, Brazil, between February and June 2022. All the pet food samples were manufactured in Brazil. The indicated ingredients on the packages of the pet food were chiefly byproducts of poultry (chicken and turkey viscera, chicken and turkey meat), other food-producing animals (such as beef, pork meat, lamb meat, animal liver, animal bones), fish, egg, and some plant-based food sources (such as rice, corn, wheat, oats, and sorghum; Table 1). Collected samples were delivered to the laboratory on the same day of collection within 2 hr for bacteriological analysis.

### 2.2. Sample Processing

Exactly 25 g of pet food samples were enriched in 225 mL of Luria-Bertani (LB) broth and homogenized for 5 min before incubation at 37°C for 24 hr. After incubation, 100 *µ*L of the broth was transferred into different microtubes which contained 900 *µ*L of tryptic soy broth (TSB) and different concentrations of meropenem (MEM) and vancomycin (VAN): Tube 1 (2 *µ*g/mL of MEM + 4 *µ*g/mL of VAN), and Tube 2 (2 *µ*g/mL of MEM). Controls were also included. Inoculated microtubes were then incubated under selective pressure at 37°C for 18–24 hr. After incubation, 10 *µ*L of broth from tubes showing turbidity (growth) were inoculated onto MacConkey agar (Oxoid, UK) and incubated at 37°C for 24 hr. Recovered pure colonies were further identified at the species level by Matrix-Assisted Laser Desorption Ionization–Time-of-Flight Mass Spectrometry (MALDI-TOF MS) using Microflex LT spectrometer and Biotyper™ 3.3 software package (Bruker Daltonics™, MA, USA), according to the manufacturer's instructions. Identified bacterial colonies were preserved at −80°C in trypticase soy broth (TSB) with 15% glycerol for further analysis.

### 2.3. Antimicrobial Susceptibility Testing

Bacterial isolates were tested for susceptibility to various antimicrobial agents using the agar dilution method [16, 17]. The following antimicrobials were tested against the isolated Gram-negative bacilli (GNB): aztreonam, ceftriaxone, ceftazidime, cefepime, ertapenem, meropenem, imipenem, amikacin, gentamicin, levofloxacin, ciprofloxacin, and tigecycline. *E. coli* ATCC® 25922™ and *Pseudomonas aeruginosa* ATCC® 27853™ were used as the quality control strains and tested within the expected ranges. Minimum inhibitory concentration (MIC) results were interpreted according to the Brazilian Committee on Antimicrobial Susceptibility Testing (BrCast)/EUCAST/criteria [16, 17].

### 2.4. Phenotypic Detection of Carbapenemase Production

All carbapenem-resistant isolates were further screened for carbapenemase production using the Blue-Carba test, as previously described [18].

### 2.5. Detection of Carbapenemase-Encoding Genes

Detection of *bla*_KPC-_like, *bla*_NDM-_like, *bla*_OXA-48_-like, *bla*_OXA-10_-like, and *bla*_OXA198_-like was done by PCR using specific primers [19–21]. The sequencing of the amplicons was done by Sanger DNA sequencing using the Kit Big-Dye Terminator Cycle Sequencer (Thermo Fisher Scientific, Foster City, USA) in the ABI 3500 Genetic Analyzer (Applied Biosystems, PerkinElmer, USA).

### 2.6. Clonal Relationship Determination by Enterobacterial Repetitive Intergenic Consensus (ERIC) PCR

The clonal relationship was initially investigated by ERIC-PCR, as previously described [22]. The amplified PCR products were electrophoresed in 1.5% agarose gel for 2 hr and visualized in the gel documentation system. The DNA band patterns were visually observed and analyzed.

### 2.7. Whole Genome Sequencing (WGS) and Genomic Data Analysis

Genomic DNA was extracted from bacterial pellets with ZymoBIOMICS DNA miniprep kit (Zymo Research) following manufacturer's instructions. The DNA purity and quantity were assessed using the Nanovue Plus (GE Healthcare) and a Qubit double-stranded DNA (dsDNA) broad range (BR) assay kit (Fisher Scientific). The sequencing libraries were prepared with the rapid barcoding sequencing kit 96 (SQK-RBK110.96; Oxford Nanopore Technologies [ONT]). Barcoded samples were loaded in a R.9.4.1 flow cell (FLO-MIN106; Oxford Nanopore Technologies Ltd.) and sequenced in a MinION Mk1C device (ONT). The sequencing data contained within the fast5 files were base-called and demultiplexed using the MinKNOW software equipped with the Dorado base-calling algorithm (version 7.0.8). This process was operated in “super-high accuracy mode” on the MinION sequencing device. Additionally, adapter sequences were trimmed, and any reads that did not meet predefined quality standards were excluded. Reads with a quality score lower than 10 were discarded. Genomes were *de novo* assembled using the workflow named “wf-denovo-assembly” (Available at: https://github.com/epi2me-labs/wf-denovo-assembly). In summary, this workflow applies the Flye v. 2.9.1 [23] for assembling and the Medaka v. 1.4.3 for polishing the contigs. Isolates were confirmed as *E. coli* by applying the SpeciesFinder 2.0 tool in Centre for Genomic Epidemiology (CGE) platform (http://www.genomicepidemiology.org) to the assembled genomes. The assembled genomes were also submitted to the comprehensive genome analysis service at BV-BRC platform (https://www.bv-brc.org/). The assembled genomes were of good quality with 3.5% contamination and 100% completeness. The MLST 2.0, PlasmidFinder 2.0, ResFinder 4.3.3, VirulenceFinder 2.0, and SerotypeFinder 2.0 databases available at the CGE were used to identify the multilocus sequence type (MLST), plasmid replicons, resistome, virulome, and serotype, respectively. A prediction filter of ≥95% and 80% were set for sequence identity and minimum length coverage thresholds, respectively. Plasmid analysis and circularization as well as genetic context analysis was performed using Geneious Prime® (version 2023.2.1).

## 3. Results

### 3.1. Frequency of Gram-Negative Bacterial Pathogens in Pet Food and Antimicrobial Susceptibility Profiles

**Figure 1:**
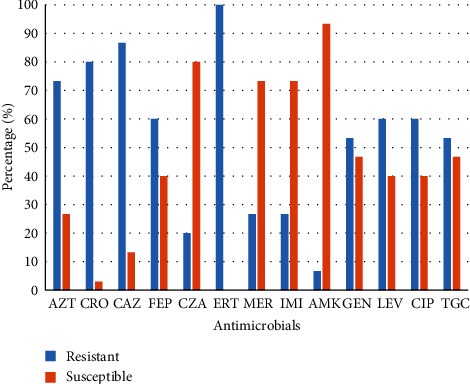
Antimicrobial resistance patterns of GNB pathogens isolated from pet food samples. Key: AZT, aztreonam; CRO, ceftriaxone; CAZ, ceftazidime; FEP, cefepime; CZA, ceftazidime/avibactam; ERT, ertapenem; MER, meropenem; IMI, imipenem; AMK, amikacin; GEN, gentamicin; LEV, levofloxacin; CIP, ciprofloxacin; TGC, tigecycline.

Results showed that 15 (17.4%) out of the 86 pet food package samples evaluated were positive for GNB: *E. coli* (*n* = 2); *Enterobacter cloacae* (*n* = 10), *Leclercia adecarboxylata* (*n* = 2), and *Cronobacter* spp. (*n* = 1) (Table 1). Only six (50%) out of the 12 different pet food brands (five cat food brands and seven dog food brands) evaluated were positive for GNB (Table 1). Ertapenem showed low activity against all GNB isolates (MIC_50_, 4 *µ*g/mL), as shown in Table 2 and Figure 1. Contrastingly, imipenem and meropenem showed *in vitro* activity against 11 (73.3%) isolates (MIC_50_, 0.5 *μ*g/mL). Aztreonam (MIC_90_, >64 *µ*g/mL) and ceftriaxone (MIC_50_, >256 *µ*g/mL) were active against 11 (73.3%) isolates. Ceftazidime (MIC_50_, 64 *µ*g/mL) and cefepime (MIC_50_, 16 *µ*g/mL) also showed activity against 13 (86.7%) and eight (53.3%) isolates, respectively. Nine (60%) isolates were resistant to fluoroquinolones (MIC_90_, >64 and 32 *μ*g/mL for ciprofloxacin and levofloxacin, respectively), while only eight isolates exhibited resistance to gentamycin (MIC_50_, 32 *μ*g/mL). In contrast, amikacin was the most active antimicrobial agent tested as 14 (93.3%) isolates were susceptible to this aminoglycoside (MIC_90_, 2 *µ*g/mL; Table 2, Figure 1). The only amikacin-resistant GNB (M17) in this study was a *L. adecarboxylata* isolate (MIC, 16 *µ*g/mL; Table 2). All isolates were resistant to tigecycline (MIC_90_, 1–4 *μ*g/mL).

### 3.2. Confirmation of Carbapenem-Resistant Bacterial Pathogens and Genetic Characterization

Only two carbapenem-resistant *E. coli* isolates (M43 and M49) were positive for Blue-Carba test (Table 2). Interestingly, the two (13.3%) carbapenem-resistant *E. coli* isolates from two different batches of brand “F” pet food sample (Table 1) harboured *bla*_KPC-2_ gene, a class A carbapenemase-encoding gene (Table 2) which was further confirmed by sequencing. The DNA sequences of the amplified *bla*_KPC_-like genes harboured by the *E. coli* isolates in our study revealed that they were 100% identical to the reference nucleotide sequence of *bla*_KPC-2_ gene (NCBI Reference Sequence: ON412784.1) deposited at the GenBank database/National Center for Biotechnology Information (NCBI) server (https://blast.ncbi.nlm.nih.gov/Blast.cgi). Interestingly, ERIC-PCR results showed that the two *bla*_KPC-2_-harbouring *E. coli* isolates belong to the same clone. These results were confirmed by WGS. However, all *E. coli* isolates were negative for *bla*_NDM-1_, *bla*_OXA-48_, *bla*_OXA-10_, and *bla*_OXA198_ carbapenemase-encoding genes.

The assembled whole genomes of the *bla*_KPC-2_-harbouring *E. coli* isolates (M43 and M49) were annotated in BV-BRC (https://www.bv-brc.org/) platform using RAST tool kit (RASTtk). Based on the annotation statistics, the two sequenced genomes were of good quality and identified as *E. coli*. Each of the two assembled genomes had, respectively: 24 and 18 contigs; total length of 5,107,014 and 5,058,972 bp; and G + C content of 50.57% and 50.59%.

Genomic analysis revealed that both M43 and M49 *E. coli* isolates also carried *tet(B*) genes in their resistome. WGS analysis also identified the *sitABCD* system encoding resistance to heavy metals and mediating the transport of manganese and iron which may contribute to oxidative stress resistance. Additionally, chromosomal mutations in *gyrA*, *gyrB*, *parC*, *parE*, *pmrA*, *pmrB*, *rrsB*, *rrsC*, *rrsH*, rpoB, *folP*, and AmpC-promoter mediating AMR were also observed. The chromosomal mutations in *gyrA*, *gyrB*, *parC*, and *parE* might be responsible for the high MICs observed for the phenotypic resistance to fluoroquinolones while the chromosomal mutations in *rrsB*, *rrsC*, and *rrsH* could be responsible for the MICs recorded for the resistance to aminoglycosides employed in this study. *In silico* analysis showed that the two *E. coli* isolates possessed several relevant virulence-encoding genes characteristic of extraintestinal pathogenic *E. coli* (ExPEC) such as: *chuA* (outer membrane hemin receptor), *kpsMIII* (ABC-type export system and group 3 capsule synthesis), *fimH* (fimbriae type I), *sitA* (iron transport protein), *air* (Enteroaggregative immunoglobulin repeat protein), *eilA* (transcriptional regulator), and *lpfA* (long polar fimbriae protein), along with an average of 484 pathogenic protein families (https://cge.food.dtu.dk/services/PathogenFinder/). Multilocus sequence typing (MLST) and serotype analyses revealed that both M43 and M49 *E. coli* isolates belong to ST648 (*adk*-92, *fumC*-4, *gyrB*-87, *icd*-96, *mdh*-70, *purA*-58, *recA*-2) and serotype O153:H2 group. Plasmidome analysis showed that both M43 and M49 harboured an IncN plasmid. Analysis of the IncN plasmids of M43 and M49 showed that they both harboured *bla*_KPC-2_ gene and also have a conserved backbone with a reference IncN plasmid (Accession number: AY046276).

**Figure 2:**
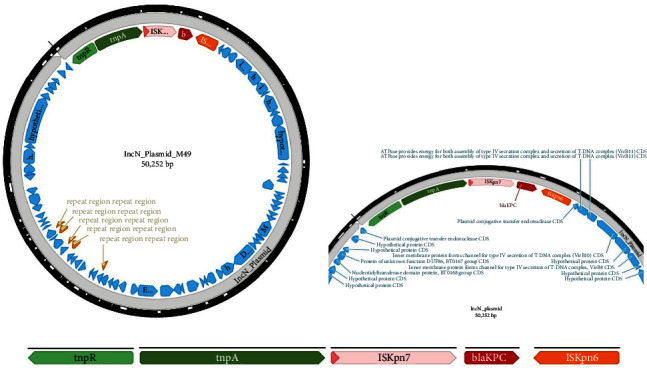
Schematics of the genetic context of *bla*_KPC-2_-carrying IncN plasmid (a,b), illustrating the *tnpR*-*tnpA*-IS*Kpn7*-*bla*_KPC−2_-IS*Kpn6* core structure (c).

The *bla*_KPC-2_ gene of M43 and M49 were both located on a Tn*3*-family transposon known as Tn*4401b* (Accession number of the reference genome: EU176014.1), and flanked upstream by IS*Kpn7*, *tnpA*, *tnpR*, and downstream by IS*Kpn6* in an ∼50.3-kb IncN plasmid to form a *tnpR-tnpA*-IS*Kpn7*-*bla*_KPC-2_-IS*Kpn6* core structure (Figure 2). The insertion sequences IS*Kpn7* and IS*Kpn6* are well-recognized putative transposition helper which are additionally flanked upstream and downstream by plasmid conjugative transfer endonuclease proteins and series of hypothetical proteins. Accessory genes, *tnpA* (a transposase) and *tnpR* (a resolvase), have also been recognized to be involved in transposition immunity.

## 4. Discussion

CRE have been recognized as critical priority pathogens by the WHO (https://www.who.int). The continued emergence and spread of CRE is a growing public health challenge in veterinary and human medicine [24]. Pet food might serve as potential sources and vehicles for the transmission of antimicrobial-resistant bacterial pathogens, including CRE to companion animals, and subsequently to humans (especially pet guardians) [25]. The zoonotic transmission risk of CRE via pet food is noteworthy as those microorganisms are recognized globally as major threats to public health.

In this study, 15 (17.4%) CRE were isolated from 86 pet food samples. These CRE isolates were also observed to be multidrug-resistant (MDR) as they exhibited resistance to at least three different antimicrobial classes. Interestingly, two (13.3%) *E. coli* isolates from different batches of pet food harboured *bla*_KPC-2_ genes which were further confirmed by sequencing. ERIC-PCR results also showed that these two *bla*_KPC-2_-harbouring *E. coli* isolates are clonally related. WGS results showed that the two *bla*_KPC-2_-harbouring *E. coli* isolates belong to the high-risk epidemiological lineage ST648 and serotype O153:H2 group. The *E. coli* ST648 is a successful evolutionary lineage and has emerged as a pandemic clone, being globally reported in humans, companion animals, and the aquatic environment [26, 27]. Our study has been able to demonstrate that pet food is a possible route for CRE transmission which has been overlooked by the scientific community. However, more studies that would holistically evaluate the transmission of clinically important bacterial pathogens (such as CRE) from contaminated pet food to companion animals (especially dogs and cats), and pet guardians/owners would be very valuable in order to comprehensively understand the epidemiological gaps concerning the acquisition of CRE within the One Health concept.

The genetic context of the *bla*_KPC-2_ showed that they were carried on a Tn*4401b* transposon in an IncN plasmid. IncN plasmids harbouring *bla*_KPC_ are known to be mobile genetic elements of worldwide epidemiological importance. Since at least 2015 to date, the circulation of *bla*_KPC-2_-harbouring IncN plasmid has been reported among *Enterobacterales* infecting humans and animals in Brazil [28], Colombia [29], USA [30], and Germany [31]. The epidemiological success of carbapenem-resistant bacterial pathogens has been attributed to plasmids carrying *bla*_KPC_-like genes that are associated with the Tn*4401* transposon [32–34]. Of interest is the IncN which has been reported to be highly disseminated, especially among international clones of *E. coli* (ST131 and ST648) [32, 33].

The prevalence of bacterial pathogens in pet food has been investigated in the United States [13] where 4.1% out of 196 frozen raw pet food samples ordered online were positive for *E. coli*. Nüesch-Inderbinen et al. [35] also reported the isolation of MDR *E. coli* and other members of the *Enterobacterales* in pet food in Switzerland. Interestingly, we also isolated two *E. coli* isolates from pet food package samples in this study. Raw pet food samples contaminated by *E. coli* were also identified as incriminating factors in a series of investigations on foodborne illnesses associated with *E. coli* in companion animals [35, 36]. A previous study in Europe (Netherlands) reported that raw pet food is more contaminated with ESBL/AmpC-producing Enterobacteriaceae than non-raw pet food products [37]. Additionally, Baede et al. [37] observed that raw pet food is an important risk factor for ESBL/AmpC shedding in household cats, and that the consistent exposure of household cats to raw pet food products appears to be linked to shedding of ESBL-positive Enterobacteriaceae, rather than mere gut colonization.

Even though there are pockets of reports on the colonization of companion animals by MDR-GNB [2, 38–40], studies on bacterial contamination of pet food are very scarce as proper attention has not been directed to this investigation. Even in Latin America, including Brazil, there are no surveillance studies on pet food contamination by bacterial pathogens, to the best of our knowledge.

Although, the CRE isolates in our study were resistant to *β*-lactams (85%), fluoroquinolones (53.3%), tigecycline (53.3%), and aminoglycosides (46.7%); however, they were highly susceptible to amikacin (93.3%). The only amikacin-resistant pathogen among the CRE was a *L. adecarboxylata* isolate (MIC, 16 *µ*g/mL). The resistance frequencies of the carbapenem-resistant bacterial pathogens in our study to *β*-lactams, fluoroquinolones, and aminoglycosides are very similar to other reports [6, 35, 41].

In the last two decades, plasmid-mediated *β*-lactamase-encoding genes in *Enterobacterales* have been widely reported in Brazil, with *bla*_KPC-2_-producing *Klebsiella pneumoniae* being the most endemic [10, 42]. Even though the pathogenesis of MDR-GNB harbouring different types of carbapenemase-encoding genes such as *bla*_KPC_-like, *bla*_NDM_-like, and *bla*_OXA-48_-like have been widely reported in humans, food-producing animals (livestock), and companion animals [2, 6, 38–40, 42]; information on pet food contamination by these MDR bacterial pathogens, especially those harbouring *bla*_KPC-2_, is very scarce. Seiffert et al. [12] reported *bla*_OXA-48_, which encodes for a class D carbapenemase in 30 analyzed pet food samples. Contrastingly, the detection of carbapenem-resistant pathogens harbouring only *bla*_KPC-2_ gene, without other types of carbapenemase-encoding genes in our study further depicts and confirms its regional dominance in Latin America, especially in Brazil.

It is interesting to know that raw materials/ingredients used in the manufacture of the pet food in our study chiefly contained byproducts of poultry (chicken and Turkey viscera, chicken and Turkey meat), other food-producing animals, fish, egg, and some plant-based food sources, according to the stated compositions on their packages. These byproducts from food-producing animals have been reported to harbor antimicrobial-resistant pathogens, including CRE [3, 9, 24, 41]. However, considering that packaged pet food samples go through sterilization techniques such as heat treatment and irradiation, it is still not clear how they were contaminated by bacterial pathogens, especially *bla*_KPC-2_-harbouring *E. coli* ST648 as reported in our study. A limitation of our study was our inability to completely ascertain the source of the pet food contamination by CRE as we did not have access to the pet food ingredients/constituents nor the production machinery. However, we suspect that contamination of the pet food might possibly be from contaminated food ingredients, machinery components/equipment, environmental contamination, or any of the production processing steps, including the final packaging of the pet food.

Reports on pet food contamination by bacterial pathogens have led to arrays of pet food recalls in the United States by the FDA [15, 43]. The consumption of these CRE-contaminated pet food by companion animals might negatively impact companion animal health and further exacerbate the zoonotic transfer potentials of these CRE from companion animals to humans [9]. Data from our study will create awareness on the significance of identifying the sources of pet food contamination as it might be an important source of clinically relevant MDR bacteria and their associated pathogenic factors. Hence, more studies are needed to elucidate the possible sources of pet food contamination.

## 5. Conclusions

In conclusion, we report for the first time in pet food, the detection of an international high-risk *bla*_KPC-2_-harbouring *E. coli* pandemic lineage ST648 in South America. The *bla*_KPC-2_ gene was inserted in the Tn*4401b* transposon carried by an IncN plasmid. The detection of CRE in packaged pet food is a critical public health problem with great significance to “One-Health” as they could serve as potential sources for the spread of MDR pathogens to companion animals, and possibly to humans through zoonotic transmission events. It is therefore imperative for the government and stakeholders in the pet food industry to reevaluate their production processes and techniques, especially proper sterilization of pet food ingredients and ensuring good Hazard Analysis Critical Control Point (HACCP) manufacturing practices to completely eradicate MDR bacterial pathogens from pet food products. This will strongly help to curb the dissemination of clinically important CRE in human and veterinary medicine.

## Figures and Tables

**Table 1:** Pet food brands, constituents, and GNB isolated.

Food brands	Batches screened	Positive batches	Food type	Major food components	Gram-negative bacteria isolated
A	9	3	Cat, dry	Chicken meat, pork meat, fish, rice, and corn	*E. cloacae* (M1) *L. adecarboxylata* (M17) *Cronobacter* spp. (M59)
B	8	0	Dog, dry	Chicken meat, fish, rice, and corn	—
C	13	5	Dog, dry	Chicken viscera and meat, chicken and pork liver, corn, and rice	*E. cloacae* (M25, M26, M27, M28) *L. adecarboxylata* (M46)
D	7	3	Cat, dry	Chicken viscera, egg, corn, fish, pumpkin, and broccoli	*E. cloacae* (M42, M50, M51)
E	6	0	Cat, dry	Poultry meat, rice, corn, and sorghum	—
F	5	2	Dog, wet	Chicken meat, beef, pork meat, wheat, corn, and animal liver	*E. coli* (M43, M49)
G	7	1	Cat, dry	Poultry viscera, beef, pork, fish, rice, and corn	*E. cloacae* (M45)
H	7	1	Dog, dry	Poultry viscera, beef, corn, wheat, and soybean	*E. cloacae* (M47)
I	5	0	Dog, dry	Chicken viscera, pork meat, and corn	—
J	6	0	Dog, dry	Poultry viscera, pork meat, and corn	—
K	7	0	Dog, dry	Poultry meat, beef, corn, and rice	—
L	6	0	Cat, dry	Chicken viscera, egg, fish, and corn	—

**Table 2:** Antimicrobial MICs for GNB isolated from pet food samples.

Isolate code	AZT	CRO	CAZ	FEP	ERT	MER	IMI	AMK	GEN	LEV	CIP	TGC	Blue-carba	*bla*_KPC-2_
*E. coli* (*n* = 2 isolates)														
M43	>64	256	32	16	256	32	64	8	8	64	>64	2	+	+
M49	>64	128	32	16	128	16	16	4	8	16	32	1	+	+
*E. cloacae* (*n* = 10 isolates)														
M1	64	1	8	≤0.125	4	0.5	8	4	4	32	>64	2	−	−
M25	2	2	64	1	8	1	0.5	2	8	<0.03	<0.03	1	−	−
M26	>64	4	64	0.25	4	0.5	2	8	32	32	>64	4	−	−
M27	16	256	64	32	4	4	2	1	0.5	32	4	1	−	−
M28	>64	>256	128	64	2	0.125	0.25	2	0.5	16	>64	2	−	−
M42	64	>256	128	32	2	0.25	2	2	32	8	>64	4	−	−
M45	64	>256	128	64	4	0.25	1	4	0.5	16	>64	4	−	−
M47	2	0.5	1	≤0.125	2	<0.06	0.5	1	0.5	<0.03	<0.03	1	−	−
M50	≤0.125	≤0.125	1	≤0.125	8	<0.06	0.25	1	0.5	<0.03	<0.03	1	−	−
M51	64	4	8	4	16	0.5	0.5	2	1	<0.03	<0.03	1	−	−
*L. adecarboxylata* (*n* = 2 isolates)														
M17	2	>256	64	1	1	<0.06	0.25	16	16	<0.03	<0.03	1	−	−
M46	>64	16	64	16	2	0.5	4	8	32	32	>64	2	−	−
*Cronobacter* spp. (*n* = 1 isolate)														
M59	>64	8	>256	16	4	8	2	4	2	0.25	0.5	1	−	−

Key: AZT, aztreonam; CRO, ceftriaxone; CAZ, ceftazidime; FEP, cefepime; ERT, ertapenem; MER, meropenem; IMI, imipenem; AMK, amikacin; GEN, gentamicin; LEV, levofloxacin; CIP, ciprofloxacin; TGC, tigecycline; +, positive; −, negative.

## Data Availability

The data that support the findings of this study are available from the corresponding author upon reasonable request.
